# Differential expression of human papillomavirus 16-, 18-, 52-, and 58-derived transcripts in cervical intraepithelial neoplasia

**DOI:** 10.1186/s12985-020-01306-0

**Published:** 2020-03-06

**Authors:** Satoshi Baba, Ayumi Taguchi, Akira Kawata, Konan Hara, Satoko Eguchi, Mayuyo Mori, Katsuyuki Adachi, Seiichiro Mori, Takashi Iwata, Akira Mitsuhashi, Daichi Maeda, Atsushi Komatsu, Takeshi Nagamatsu, Katsutoshi Oda, Iwao Kukimoto, Yutaka Osuga, Tomoyuki Fujii, Kei Kawana

**Affiliations:** 1 Department of Obstetrics and Gynecology, Graduate School of Medicine, The University of Tokyo, Tokyo, Japan; 2 Department of Gynecology, Tokyo Metropolitan Cancer and Infectious Diseases Center, Komagome Hospital, Tokyo, Japan; 3 Department of Public Health, Graduate School of Medicine, The University of Tokyo, Tokyo, Japan; 4 Pathogen Genomics Center, National Institute of Infectious Diseases, Tokyo, Japan; 5 Department of Obstetrics and Gynecology, Keio University School of Medicine, Tokyo, Japan; 6 Department of Reproductive Medicine, Chiba University Graduate School of Medicine, Chiba, Japan; 7 Department of Cellular and Organ Pathology, Graduate School of Medicine, Akita University, Akita, Japan; 8 Department of Clinical Genomics, Graduate School of Medicine, Osaka University, Osaka, Japan; 9 Department of Obstetrics and Gynecology, Nihon University School of Medicine, Tokyo, Japan

**Keywords:** Human papillomavirus, Cervical intraepithelial neoplasia, Viral transcripts

## Abstract

**Background:**

Human papillomavirus (HPV) infection is a primary cause of cervical cancer. Although epidemiologic study revealed that carcinogenic risk differs according to HPV genotypes, the expression patterns of HPV-derived transcripts and their dependence on HPV genotypes have not yet been fully elucidated.

**Methods:**

In this study, 382 patients with abnormal cervical cytology were enrolled to assess the associations between HPV-derived transcripts and cervical intraepithelial neoplasia (CIN) grades and/or HPV genotypes. Specifically, four HPV-derived transcripts, namely, oncogenes *E6* and E6*, *E1^E4*, and viral capsid protein *L1* in four major HPV genotypes—HPV 16, 18, 52, and 58—were investigated.

**Results:**

The detection rate of E6/E6* increased with CIN progression, whereas there was no significant change in the detection rate of *E1^E4* or *L1* among CIN grades. In addition, we found that *L1* gene expression was HPV type-dependent. Almost all HPV 52-positive specimens, approximately 50% of HPV 58-positive specimens, around 33% of HPV 16-positive specimens, and only one HPV18-positive specimen expressed *L1*.

**Conclusions:**

We demonstrated that HPV-derived transcripts are HPV genotype-dependent. Especially, expression patterns of *L1* gene expression might reflect HPV genotype-dependent patterns of carcinogenesis.

## Background

Human papillomavirus (HPV) infection is a common sexually transmitted disease, with approximately 50–80% of sexually active adolescents being infected within 2–3 years of initiating intercourse [1]. Most HPV infections are latent by immune regression, while about 10% of the infections are proliferative, which is associated with cervical cancer development [2]. The International Agency for Research on Cancer divided the HPV genotypes into the following groups according to their carcinogenesis: the highly carcinogenic Group 1 (HPVs 16, 18, 31, 33, 35, 39, 45, 51, 52, 56, 58, and 59); the probably carcinogenic Group 2A (HPV 68); and the possibly carcinogenic Group 2B (HPVs 26, 30, 34, 53, 66, 67, 69, 70, 73, 82, 85, and 97) [3]. Continuous expression of HPV *E6* and *E7* oncogenes, mainly caused by integration of the HPV genome into the human genome, is critical in cervical cancer progression [4]. High-risk HPV *E6* and *E7* are likely to transform CIN lesion to cancer. Especially, HPV 16 and 18 are the most carcinogenic. The prevalence of HPV 16 and 18 in cervical cancer and cervical intraepithelial neoplasia (CIN) are quite different from other high-risk HPV. About 50 and 15% of cervical cancer are positive for HPV 16 and 18, whereas about 40% and 3–7% of high-grade CIN (CIN2/3) are positive, respectively [5, 6]. Other data show that the rate of progression of HPV 16- or 18-infected cervical epithelium to CIN3 or more is around 15% at 10 years post-infection, which is much higher compared to other HR-HPV [7]. Further, HPV 18 is likely to integrate the viral genome into the host genome compared to HPV 16 [8]. Furthermore, there are HPV type-dependent features among cancer histological types. Most HPV 16-positive cancers are squamous cell carcinomas, whereas around 50% of HPV 18-positive cancers are adenocarcinomas [5].

HPV generates numerous viral transcripts via differential RNA splicing. For example, at least 13 transcripts are derived from eight HPV genes in HPV 16-infected W12E cells [9]. There are six genes (*E6*, *E7*, *E1*, *E2*, *E4*, and *E5*) located in the early region of the HPV genome, and two genes (*L1* and *L2*) in the late region. Expression of these genes is altered during epithelial differentiation and/or CIN progression. *E6* and *E7* are oncogenes encoding proteins that suppress p53 and pRb activation, respectively [10]. E6* is a splicing isoform of *E6*, which is the main *E6* isoform in cervical cancer, and might facilitate *E7* expression [11]. Although the roles of *E1^E4* are not explicitly defined, *E1^E4* is considered to be associated with viral replication [12]. The *L1* and *L2* proteins are components of the viral capsid and are associated with HPV infection [13].

The expression patterns of HPV-derived transcripts vary depending on CIN grade. For example, the expression of *E6* and *E7* is higher in high-grade squamous intraepithelial lesions (high-grade SILs) than in low-grade SILs [14]. In contrast, expression of the *L1* protein is lower in high-grade SILs [15, 16]. The expression patterns of HPV-derived transcripts also differ among HPV genotypes. For example, among *E6* isoforms, HPV 18 cancers exhibit significantly higher ratios of the non-spliced isoform of *E6* oncoprotein than HPV 16 cancers [17]. Furthermore, Griffin et al. demonstrated that CIN3 with HPV 18 exhibited no *E4* protein expression [18].

In this study, we analyzed each HPV-derived transcript to gain a better understanding of HPV genotype-dependent carcinogenesis. To represent the viral life cycle, we focused on the expression levels of HPV-derived transcripts E6/E6*, *E1^E4*, and L1. E6/E6* are oncogenes regulated by the early promoter, the *E1^E4* splicing site relates to both early and late gene expression and can contribute to viral replication, and *L1* expression is observed in the late phase of viral differentiation, which is regulated by the late promoter [19]. In addition to HPV 16 and 18, we focused on HPV 52 and 58, which are highly prevalent in East Asia [20].

## Methods

### Patients and sample collection

All experimental procedures were approved by the institutional review boards of The University of Tokyo (approval number: G10082), Keio University (approval number: 2015–388), Chiba University (approval number: 560), Akita University (approval number: 2174), the National Institute of Infectious Diseases (approval number: 659), and Nihon University (approval number: 234–0), and signed informed consent for the use of tissues was obtained from each participant.

In total, 382 patients with cervical cytological abnormality who were admitted to the University of Tokyo, Chiba University, or Keio University between February 2016 and December 2017 were enrolled. Cervical tissues were obtained from biopsy under colposcopic examination. Samples were stored at − 80 °C until analysis.

### Variables

Clinical data, such as age, gravidity, smoking history, usage of steroids or immunosuppressants, and time from first detection of abnormal cytology, were obtained by a medical interview. Histological results were classified into three CIN grades: CIN1, CIN2, and CIN3. Diagnosis was confirmed by a pathologist at Akita University.

The results of the HPV genotyping in cervical samples were recorded. It was permitted to assign multiple genotypes to a single patient. In this study, on the basis of the classification of the International Agency for Research on Cancer, we defined HPVs classified in Group 1 (HPV 16, 18, 31, 33, 35, 39, 45, 51, 52, 56, 58, and 59) as “high-risk HPVs (hrHPVs)” [3]. Of these, HPVs 16, 18, 52, and 58 were separately categorized. hrHPVs other than HPV 16, 18, 52, and 58 were classified as “other hrHPVs.”

### HPV genotyping

DNA was extracted from cervical specimens using the Tissue Genomic DNA Extraction Mini Kit (Favorgen Biotech Corp., Ping-Tung, Taiwan) at The University of Tokyo. HPV genotyping was performed at the National Institute of Infectious Diseases using the PGMY-CHUV assay method as described previously [21]. Briefly, standard PCR was conducted using the PGMY09/11 *L1* consensus primer set and human leukocyte antigen-DQ (HLADQ) primer sets. Subsequently, reverse blotting hybridization was performed. Heat-denatured PCR amplicons were hybridized to probes specific for 31 HPV genotypes and HLA-DQ references [22].

### Primer design and standard plasmid

PCR primers were designed using Primer-Blast (NCBI) in reference to PaVE, the papilloma virus genome database. The following criteria were considered when designing the primer pairs: (1) each primer should be 19–23 bp in length, and (2) the amplicon should be between 70 and 260 bp in length. The primer design is shown in S1 Fig. Plasmid standards (Eurofin Scientific, Luxembourg City, Luxembourg) were used to derive standard curves for absolute quantification.

### RNA extraction and quantitative real-time PCR (qRT-PCR)

Total RNA was extracted from cervical specimens using an miRNeasy Mini Kit (Qiagen, Hilden, Germany) after DNase treatment using the RNase-Free DNase Set (Qiagen, Hilden, Germany) at The University of Tokyo. Extracted RNA was reverse-transcribed using the SuperScript III First-Strand Synthesis System for RT-PCR (Life Technologies, Carlsbad, CA, USA) according to the manufacturer’s instructions. To assess mRNA expression levels, qRT-PCR was performed using a Light Cycler 480 system (Roche Diagnostics GmbH, Mannheim, Germany) with 1 μL of cDNA. Expression of HPV-derived transcripts was normalized to that of *GAPDH* mRNA as an internal standard. The normalized copy number was calculated as follows: normalized copy number = copy number/2^^[30 - GAPDH Cp value]^. Primer pairs for amplification of *GAPDH* and each HPV-derived transcript are shown in Table 1. PCR conditions were as follows: 45 cycles at 95 °C for 10 s, 62 °C for 10 s, and 72 °C for 18 s. All PCR reactions were assessed using melting curve analysis.

**Table 1:** Primer pairs used for qRT-PCR

Target	Direction	Sequence	Product size (bp)	Genome position
GAPDH	Forward	GAAAGGTGAAGGTCGGAGTC	227	
	Reverse	GAAGATGGTGATGGGATTTC		
HPV 16 *E6*	Forward	AGCGACCCAGAAAGTTACCAC	260	123–143
	Reverse	GTTGTATTGCTGTTCTAATGTTG		382–360
HPV 16 E6*	Forward	AGCGACCCAGAAAGTTACCAC	114	123–143
	Reverse	TTAATACACCTCACGTCGC		418–409 + 226–217
HPV 16 *E1^4*	Forward	CCTGCAGCAGCAACGAAGTATC	218	874–880 + 3358–3372
	Reverse	TTGGTCGCTGGATAGTCGTC		3479–3460
HPV 16 *L1*	Forward	GTCTCTTTGGCTGCCTAGTG	89	5641–5660
	Reverse	TGCGTGCAACATATTCATCCG		5729–5709
HPV 18 *E6*	Forward	AACACGGCGACCCTACAAG	248	125–143
	Reverse	ATGTGTCTCCATACACAGAGTC		372–351
HPV 18 E6*	Forward	AACACGGCGACCCTACAAG	120	125–143
	Reverse	ACCGCAGGCACCTCTGTAAG		426–416 + 233–225
HPV 18 *E1^4*	Forward	GATCCAGAAGTACCAGTGAC	194	920–929 + 3434–3443
	Reverse	GAGAAGTGGGTTGACAGGTC		3617–3598
HPV 18 *L1*	Forward	TCCTTCTGTGGCAAGAGTTGT	123	5657–5677
	Reverse	CCACCTGCAGGAACCCTAAAA		5779–5759
HPV 52 *E6*	Forward	TTTGAGGATCCAGCAACAC	197	105–123
	Reverse	TAGGCACATAATACACACGCC		302–282
HPV 52 E6*	Forward	TTTGAGGATCCAGCAACAC	128	105–123
	Reverse	GACAAATTATACATCTCTCTTCG		510–502 + 216–224
HPV 52 *E1^4*	Forward	AGGACCCTGAAGTAACGAAG	150	868–879 + 3345–3352
	Reverse	CTGGAGTCTGTGACGTCTGG		3482–3463
HPV 52 *L1*	Forward	ACTGTGTACCTGCCTCCTGTA	72	5670–5690
	Reverse	GATGCTTGTGCGAGACACAT		5741–5722
HPV 58 *E6*	Forward	GAAACCACGGACATTGCATG	254	130–149
	Reverse	GTGTTTGTTCTAATGTGTCTCC		383–362
HPV 58 E6*	Forward	GAAACCACGGACATTGCATG	109	130–149
	Reverse	CAAATAATACATCTCAGATCGC		515–510 + 232–223
HPV 58 *E1^4*	Forward	GACCCTGAAGTGATCAAATATC	127	889–898 + 3358–3372
	Reverse	GTGTTGTCTCTGGAGTCTGG		3471–3452
HPV 58 *L1*	Forward	CCTCCTGTGCCTGTGTCTAA	104	5682–5700
	Reverse	GGATTGCCAACAGCCAAAAGT		5785–5765

Primer information, such as sequence, product size, and genome position of the primer pairs was summarized

### Statistical analysis

Categorized clinical features such as gravidity, smoking history, usage of steroids or immunosuppressants according to HPV categories and CIN grades were evaluated using the Analysis of Variance. Other clinical features such as age and time from first detection of abnormal cytology and the expression levels of each transcriptome according to HPV types and CIN grades were analyzed using a Steel-Dwass test. The relationship between each HPV infection and positive ratio of each transcriptome was evaluated using the Cochran-Armitage trend test. Statistical analyses were performed with the JMP Pro software (13.0.0). *p* < 0.05 was considered significant. If the viral gene copy number was greater than 10 copies/L, the sample was considered positive for gene expression.

## Results

### HPV prevalence of four major genotypes

**Fig. 1:**
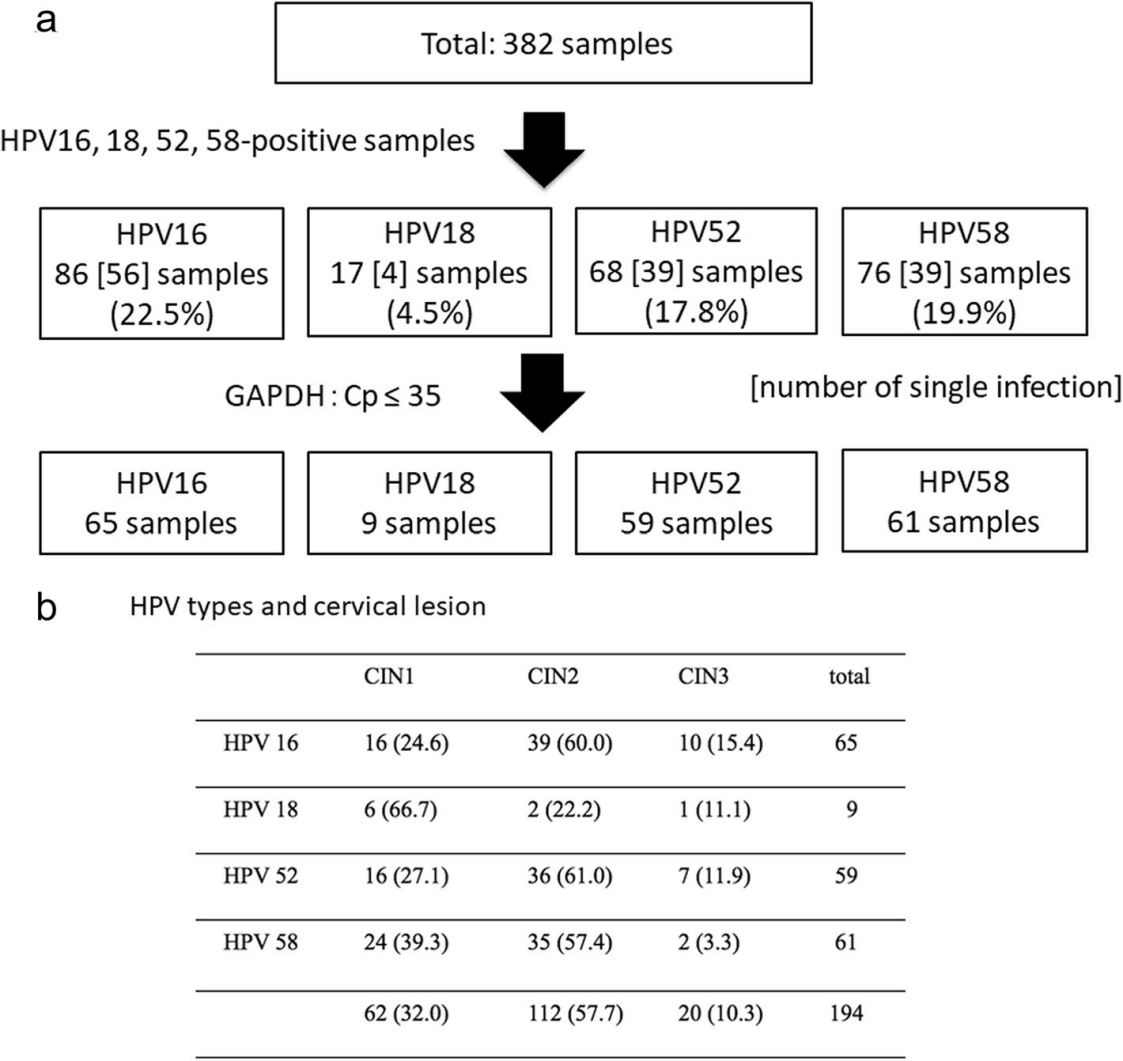
CIN grades and prevalence of four major HPV genotypes. **a** Flow chart of patient enrollment; **b** CIN grades and HPV infection genotypes of the patients enrolled in this study

For the 382 patients with cervical cytological abnormality enrolled in the study, CIN grades and infected HPV types are summarized in Fig. 1. HPV 16 was detected in 86 (22.5%) samples, HPV 18 was detected in 17 (4.5%) samples, HPV 52 was detected in 68 (17.8%) samples, HPV 58 was detected in 76 (19.9%) samples, and other hrHPVs were detected in 83 (21.7%) samples. Samples infected with a single genotype included 56 (65.1%) HPV 16-positive samples, 4 (23.5%) HPV 18-positive samples, 39 (57.3%) HPV 52-positive samples, and 39 (51.3%) HPV 58-positive samples (Fig. 1). The ages of patients with each HPV genotype were significantly different, whereas no significant differences were found in gravidity, smoking history, usage of steroids or immunosuppressants, or time from first detection of abnormal cytology among patients with each HPV genotype (Tables 2 and 3).

**Table 2:** Clinical features according to HPV type

	HPV 16	HPV 18	HPV 52	HPV 58	Other hrHPVs	Negative	*p*-value
Age (years)	37 ± 0.9	39 ± 3.6	38 ± 0.8	39 ± 1.0	44 ± 1.2	40 ± 1.7	0.001^*1^
Months since diagnosis	40 ± 5.2	38 ± 10.0	36 ± 4.4	39 ± 5.1	37 ± 7.2	37 ± 7.0	0.95^*1^
Smoking (%)	15/55 (27.3)	3/10 (30.0)	14/56 (25.0)	19/56 (28.4)	16/72 (22.2)	7/39 (18.0)	0.57^*2^
Parity ≥1 (%)	23/57 (40.3)	3/10 (30.0)	23/56 (41.1)	22/57 (38.6)	26/74 (35.1)	15/40 (37.5)	0.97^*2^
Steroid use (%)	0/59 (0.0)	0/10 (0.0)	2/57 (3.5)	2/58 (3.5)	2/74 (2.7)	1/36 (2.8)	0.56^*2^

Clinical features were summarized according to HPV categorize. HPVs 16, 18, 31, 33, 35, 39, 45, 51, 52, 56, 58, 59, and 68 were classified as hrHPVs. Of these, HPV 16, 18, 52, and 58 were categorized separately. hrHPVs other than HPVs 16, 18, 52, and 58 were classified as “other hrHPVs.” Patients who were not infected with any hrHPVs were referred to as “no hrHPVs” patients. Statistical analysis was performed using a Steel-Dwass test (*1) and the Analysis of Variance (*2)

*HPV* human papillomavirus

**Table 3:** Clinical features according to cervical lesion grade

	CIN1	CIN2	CIN3	***p***-value
Age (years)	36 ± 1.1	38 ± 0.6	32.5 ± 2.0	0.28^*1^
Months since diagnosis	23 ± 4.3	26 ± 3.9	9 ± 9.9	0.07^*1^
Smoking (%)	10/55 (18)	31/93 (33)	6/19 (32)	0.12^*2^
Parity ≥1 (%)	21/56 (38)	36/96 (38)	10/19 (53)	0.36^*2^
Steroid use (%)	2/57 (3.5)	2/98 (2.0)	0/19 (0.0)	0.67^*2^

Clinical features were summarized according to cervical lesion grade. Statistical analysis was performed using a Steel-Dwass test (*1) and the Analysis of Variance (*2)

*CIN* cervical intraepithelial neoplasia

### Differential E6/E6* expression in HPV-positive specimens

**Fig. 2:**
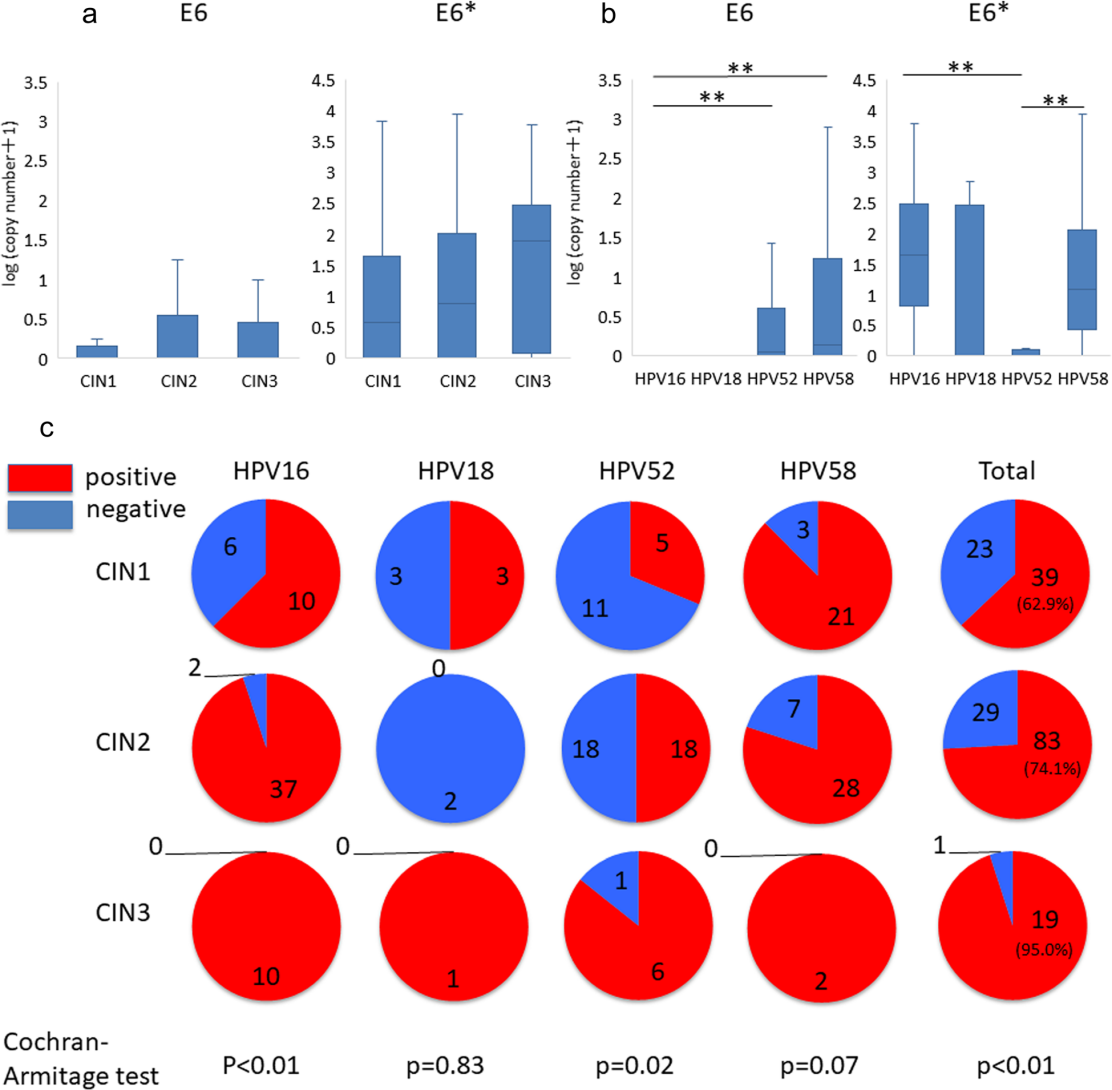
Expression of HPV *E6* and/or E6* genes. **a** Copy number of *E6* and E6* genes in specimens with different CIN grades. Statistical analysis was performed using a Steel-Dwass test. ** indicates *p* < 0.01. **b** Copy number of *E6* and E6* genes in specimens with HPV16, 18, 52, and 58 infection. Statistical analysis was performed using a Steel-Dwass test. ** indicates *p* < 0.01. **c** Detection rate of *E6* and/or E6* gene in each genotype stratified by CIN grade. Statistical analysis was performed using a Cochran-Armitage test

Expression levels of oncogene *E6* and its isoform E6* were evaluated in each sample. Although there was no difference in the expression levels of *E6* or E6* among CIN grades (Fig. 2a and b), the detection rate of *E6* and/or E6* increased with severity of the CIN grade (Cochran-Armitage test, *p* < 0.01, Fig. 2c). In terms of HPV genotype-dependent analysis, *E6* expression was lowest and E6* expression was highest in HPV 16 positive-specimens among the four HPV genotypes. In HPV 18-positive specimens, although no significant differences compared to other HPV genotypes were observed, possibly due to the small sample size, *E6* and E6* expression patterns were similar to HPV 16-positive specimens. Conversely, in HPV 52-positive specimens, *E6* expression was higher than E6*, while comparable expression levels of both *E6* and E6* were observed in HPV 58-positive specimens (Fig. 2b).

### Differential E1^E4 expression in HPV-positive specimens

**Fig. 3:**
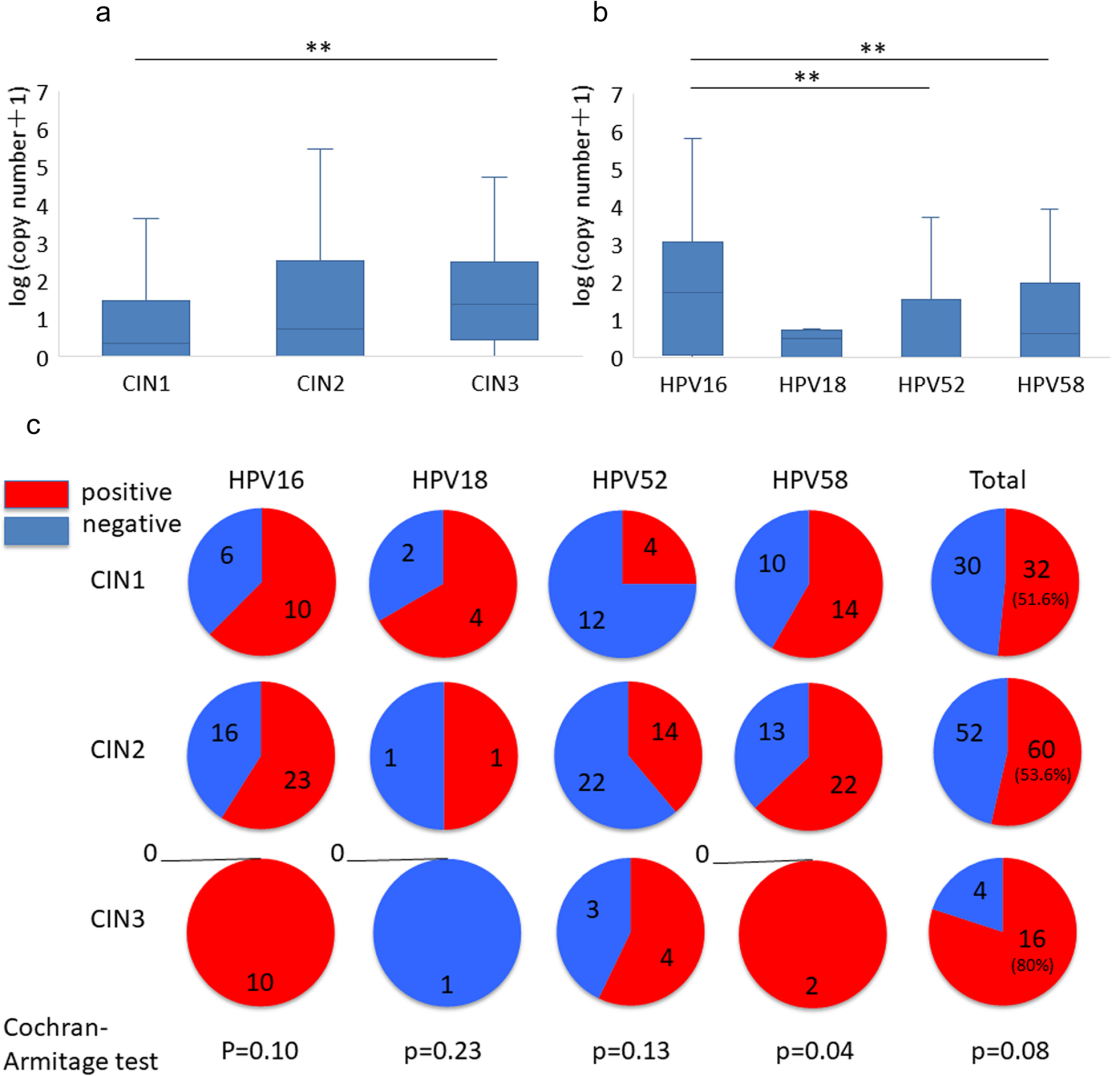
Expression of HPV *E1^E4* gene. **a** Copy number of the *E1^E4* gene in specimens with different CIN grades. Statistical analysis was performed using a Steel–Dwass test. ** indicates *p* < 0.01. **b** Copy number of the *E1^E4* gene in specimens with HPV16, 18, 52, and 58 infection. Statistical analysis was performed using a Steel–Dwass test. * indicates normalized copy number. ** indicates *p* < 0.01. **c** Detection rate of *E1^E4* gene in each genotype stratified by CIN grade. Statistical analysis was performed using a Cochran-Armitage test

Subsequently, expression of the *E1^E4* splicing site was evaluated. Positivity of this site is related to both early and late gene expression [19]. First, we compared *E1^E4* expression levels across CIN grades and found its highest expression in CIN3 (Fig. 3a). The detection rate of *E1^E4* tended to increase with CIN progression in HPV 16-, 52-, and 58-positive specimens (Cochran-Armitage test, *p* = 0.10, *p* = 0.13, and *p* = 0.04, respectively) (Fig. 3c). HPV genotype-dependent analysis revealed that *E1^E4* expression levels were highest in HPV 16-positive specimens and lowest in HPV 18-positive specimens (Fig. 3b). Further, the detection rate of *E1^E4* was higher in HPV 16- and 58-positive specimens than HPV 52-positive specimens (Fig. 3c).

### HPV type-dependent expression of major capsid protein *L1*

**Fig. 4:**
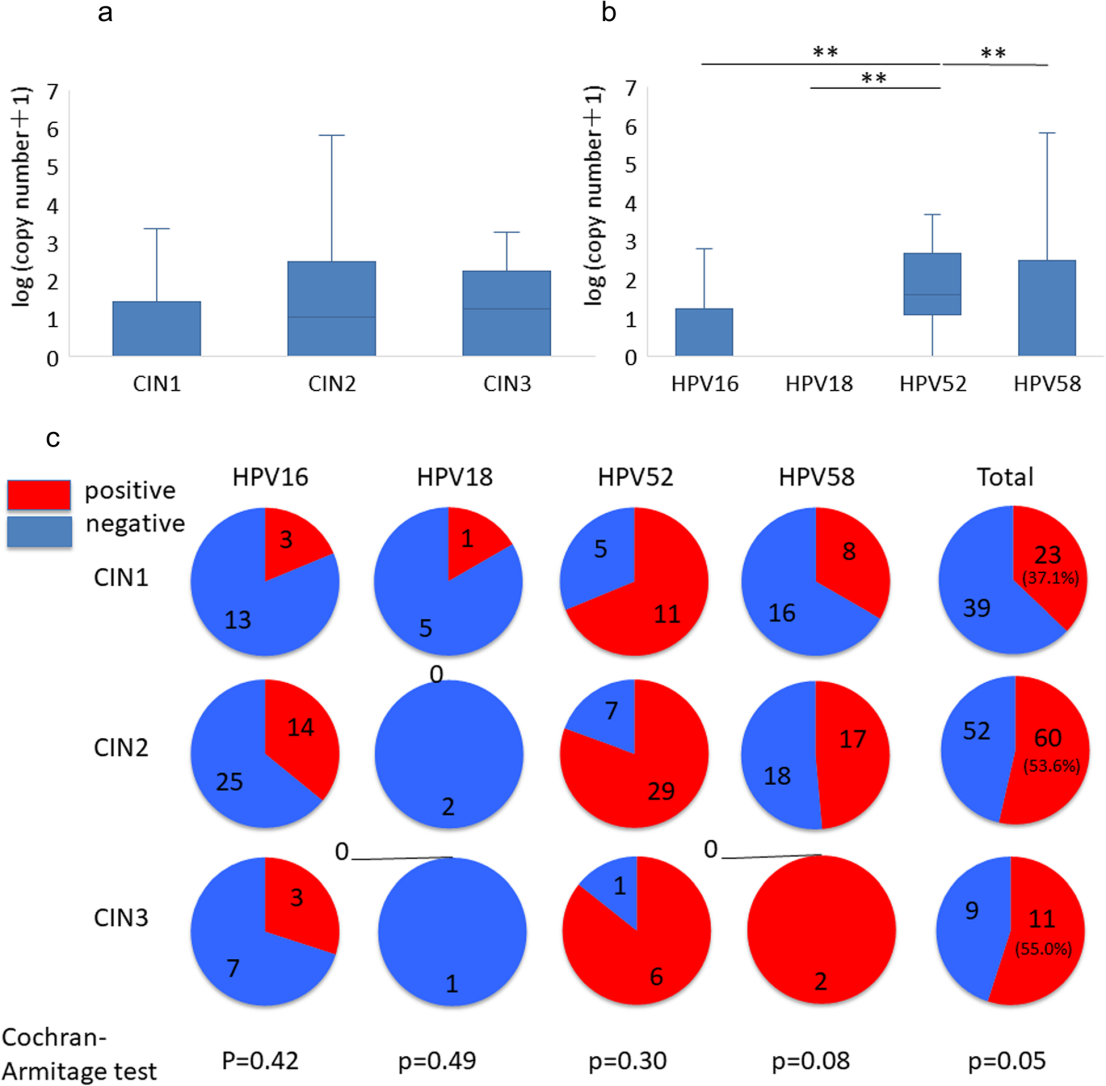
Expression of the HPV *L1* gene. **a** Copy number of the *L1* gene in specimens with different CIN grades. Statistical analysis was performed using a Steel–Dwass test. ** indicates *p* < 0.01. **b** Copy number of *L1* gene in specimens with HPV16, 18, 52, and 58 infection. Statistical analysis was performed using a Steel–Dwass test. ** indicates *p* < 0.01. **c** Detection rate of *L1* gene in each genotype stratified by CIN grade. Statistical analysis was performed using a Cochran-Armitage test

Further, expression levels of the *L1* gene, which encodes a major capsid protein, was assessed. There was no difference in *L1* expression among CIN grades (Fig. 4a). Comparison of *L1* gene expression among HPV genotypes revealed the highest *L1* expression in HPV 52-positive specimens, followed by HPV 58-positive specimens, and there was almost no *L1* expression in HPV 18-positive specimens (Fig. 4b). Additionally, *L1* expression was HPV type-dependent, in which nearly 100% of HPV 52-positive specimens, around 50% of HPV 58-positive specimens, approximately 33% of HPV 16-positive specimens, and almost 0% of HPV 18-positive specimens expressed *L1* (Fig. 4c).

Since *L1* expression is a hall mark of viral production and viral production is typically accompanied by epithelial differentiation, we performed immunohistochemistry analysis of KRT10 and KRT13 to investigate epithelial differentiation. However, there was no difference in KRT10 and KRT13 expression among samples with each HPV genotype (S2 Fig).

## Discussion

We performed an in-depth analysis of HPV-derived transcript levels according to HPV genotype and CIN grade. The detection rate of E6/E6* increased with CIN progression, which is consistent with a previous study [23], whereas there was no significant change in the detection rate of *E1^E4* or *L1* among CIN grades. Furthermore, type-dependent analysis revealed that expression patterns of HPV-derived transcripts were HPV genotype-dependent.

Interestingly, *L1* expression level was lowest in HPV 18-positive specimens among the four genotype groups. HPV 18 is one of the most carcinogenic genotypes among HR-HPV [24], and frequently observed in young aged cervical cancer [1]. In addition, around 40% of cervical adenocarcinoma is caused by HPV 18 infection [5]. *L1* protein, a major component of the viral capsid, is a hallmark of viral production accompanied with cellular differentiation. Therefore, low level or lack of *L1* expression in HPV 18-positive specimens may be associated with the loss of cellular differentiation and non-proliferative HPV infection, suggesting that stratified epithelium differentiation is not necessary for the HPV 18 genome replication and maintenance of HPV 18-related carcinogenesis. Our results added a new insight on HPV 18-related carcinogenesis from the aspect of HPV-derived transcripts. Other than loss of cellular differentiation, expression of the HPV *L1* capsid protein disappears when HPV DNA is integrated into the host genome. Viral genome integration occurs earlier in HPV 18-positive cervical cells than in HPV 16-positive cells [8]. Loss of cell differentiation and viral genome integration from the early stage of CIN might be associated with rapid cancer development of HPV 18-infected CINs.

Conversely, *L1* expression was highest in HPV 52, even in high-grade SIL (CIN2 and CIN3). Usually, *L1* gene expression decreases as the CIN grades progress due to the lack of cellular differentiation [15, 16]. In contrast to HPV 18, the high expression of the *L1* gene in HPV 52-positive specimens, even in high-grade SILs, may indicate that proliferative HPV infection accompanied with cellular differentiation may be maintained in HPV 52-positive lesions. In this study, we could not identify the human differentiation markers reflecting HPV 52-positive lesions; further studies are needed to identify human gene expression profiles that can distinguish the expression patterns of HPV-derived transcriptomes. Furthermore, the high level of *L1* gene expression in HPV 52-positive CIN3 suggests that viral genome integration occurs in the late stage of CIN progression in these samples. Combined with the previously reported epidemiological findings, i.e. frequent observation of HPV 52 in CIN lesions compared to cancer lesions [5], high *L1* expression represents the long-term persistence of HPV 52-related CINs.

This study has several limitations. First, regarding the analysis of *E1^E4* gene expression, we only evaluated the expression level of the *E1^E4* splicing site. Therefore, it is difficult to precisely demonstrate the significance of *E1^E4* expression on the biology of viral replication or cancer development. Second, the sample size of the HPV 18-positive specimen was small. Therefore, further study using a large sample size is warranted to confirm our results. Third, this study is a cross-sectional study and does not investigate prognosis of CIN patients. As such, a prospective cohort study is needed to investigate whether expression of these HPV-derived transcripts can be biomarkers of CIN progression or regression.

## Conclusions

In this study, we investigated the expression of three HPV-derived transcripts downstream of early, early/late, and late promoters in CIN lesions. Their expression patterns differed among HPV genotypes. In particular, *L1* gene expression levels were lowest in HPV 18, while highest in HPV 52, suggesting HPV type dependence of HPV-derived carcinogenesis and viral maintenance in the cervical epithelium.

## Supplementary information

**Additional file 1: Figure S1.** Primer designs for each transcriptome. The primer designs for *E6*, E6*, *E1^E4*, and *L1* were summarized.

**Additional file 2: Figure S2.** Keratin (KRT)10 and KRT13 immunohistochemistry of cervical lesions. (a) Expression of KRT10 and KRT13 in CIN1 and 3. Specimens were stained with anti-KRT10 antibody (GNT, Irvine, CA, USA) and anti-KRT13 antibody (GNT, Irvine, CA, USA) according to the manufacturer’s instructions. Bars indicate 100 μm. (b) Detection rate of KRT10 and KRT13 in each genotype stratified by CIN grade.

## Data Availability

https://datadryad.org/review?doi=doi:10.5061/dryad.r5d48s1

## Abbreviations

CIN: Cervical intraepithelial neoplasia
HLA-DQ: Human leukocyte antigen-DQ
HPV: Human papillomavirus
PaVE: Papillomavirus episteme
SILs: Squamous intraepithelial lesions
