# Supplementary figures and images for: Differential expression of human papillomavirus 16-, 18-, 52-, and 58-derived transcripts in cervical intraepithelial neoplasia
Source: Virol J. 2020 Mar 6;17:32. doi: 10.1186/s12985-020-01306-0 (PMC7060624; doi:10.1186/s12985-020-01306-0)

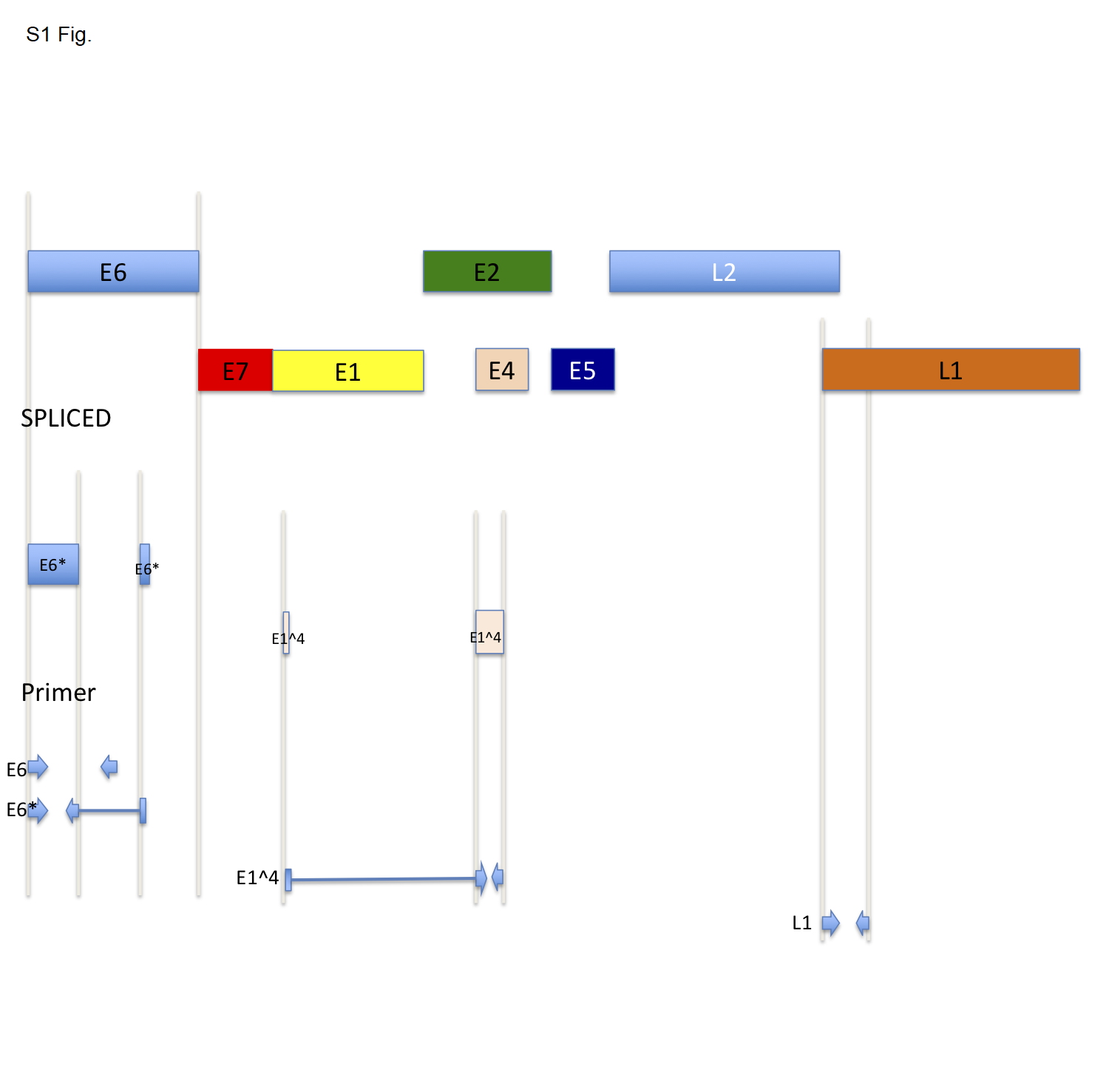

Supplement: Supplementary file 1 — Additional file 1: Figure S1. Primer designs for each transcriptome. The primer designs for E6, E6*, E1^E4, and L1 were summarized. [file 12985_2020_1306_MOESM1_ESM.tiff]

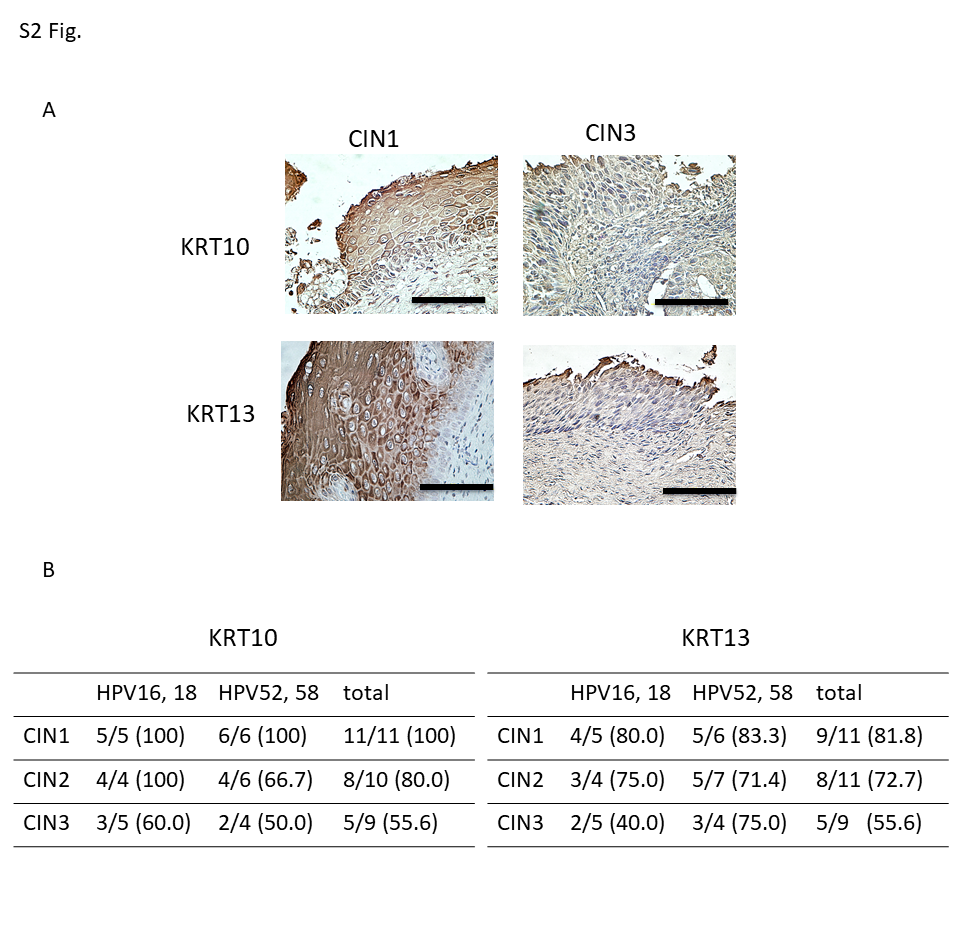

Supplement: Supplementary file 2 — Additional file 2: Figure S2. Keratin (KRT)10 and KRT13 immunohistochemistry of cervical lesions. (a) Expression of KRT10 and KRT13 in CIN1 and 3. Specimens were stained with anti-KRT10 antibody (GNT, Irvine, CA, USA) and anti-KRT13 antibody (GNT, Irvine, CA, USA) according to the manufacturer’s instructions. Bars indicate 100 μm. (b) Detection rate of KRT10 and KRT13 in each genotype stratified by CIN grade. [file 12985_2020_1306_MOESM2_ESM.tif]
